# A Peek into Pandora’s Box: COVID-19 and Neurodegeneration

**DOI:** 10.3390/brainsci12020190

**Published:** 2022-01-30

**Authors:** Abhishek Chandra, Ashu Johri

**Affiliations:** Independent Researcher, New York, NY 10065, USA; abchandra2010@gmail.com

**Keywords:** COVID-19, SARS-CoV-2, neurodegeneration, therapeutics, mitochondria, biomarker

## Abstract

Ever since it was first reported in Wuhan, China, the coronavirus-induced disease of 2019 (COVID-19) has become an enigma of sorts with ever expanding reports of direct and indirect effects of the severe acute respiratory syndrome coronavirus 2 (SARS-CoV-2) on almost all the vital organ systems. Along with inciting acute pulmonary complications, the virus attacks the cardiac, renal, hepatic, and gastrointestinal systems as well as the central nervous system (CNS). The person-to-person variability in susceptibility of individuals to disease severity still remains a puzzle, although the comorbidities and the age/gender of a person are believed to play a key role. SARS-CoV-2 needs angiotensin-converting enzyme 2 (ACE2) receptor for its infectivity, and the association between SARS-CoV-2 and ACE2 leads to a decline in ACE2 activity and its neuroprotective effects. Acute respiratory distress may also induce hypoxia, leading to increased oxidative stress and neurodegeneration. Infection of the neurons along with peripheral leukocytes’ activation results in proinflammatory cytokine release, rendering the brain more susceptible to neurodegenerative changes. Due to the advancement in molecular biology techniques and vaccine development programs, the world now has hope to relatively quickly study and combat the deadly virus. On the other side, however, the virus seems to be still evolving with new variants being discovered periodically. In keeping up with the pace of this virus, there has been an avalanche of studies. This review provides an update on the recent progress in adjudicating the CNS-related mechanisms of SARS-CoV-2 infection and its potential to incite or accelerate neurodegeneration in surviving patients. Current as well as emerging therapeutic opportunities and biomarker development are highlighted.

## 1. Introduction

At the end of 2019, the whole world was caught off guard by the sudden and unprecedented emergence of severe acute respiratory syndrome coronavirus 2 (SARS-CoV-2) infections, which caused coronavirus-induced disease of 2019 (COVID-19). Although respiratory distress is the main feature of the disease, other symptoms have also been well described as mentioned later in this review. Thus far, several therapeutics have been tested, some of which are summarized in Table 1, however, there is still no cure and clinical care is limited to symptomatic treatment on a case-by-case basis. COVID-19 vaccines have been developed and more are in development, but none of them are 100% effective and we are still learning about the duration of efficacy for the various vaccines and the need for booster shots due to the regular emergence of new variants (https://www.who.int/news/item/22-12-2021-interim-statement-on-booster-doses-for-covid-19-vaccination---update-22-December-2021; accessed on 28 January 2022 [1,2,3,4]). The longer persisting adverse effects resulting from this viral infection are still not fully defined yet. This review is an attempt to compile the current knowledge on SARS-CoV-2 and COVID-19 and also briefly describe the association of this virus with the nervous system. We begin with a brief description of general facts about COVID-19 and SARS-CoV-2.

### 1.1. Chronology of COVID-19

The original SARS-CoV outbreak occurred in 2002, while severe acute respiratory syndrome coronavirus-2 (SARS-CoV-2) was first reported to the World Health Organization (W.H.O.) on 31 December 2019. A cluster of cases of “viral pneumonia” were first reported in Wuhan, the People’s Republic of China, which turned out to be caused by SARS-CoV-2 [5]. W.H.O. termed this disease as COVID-19 on 11 February 2020, and declared it a global pandemic on 11 March 2020 [6]. As of January 2022, almost all countries have been infected by this highly contagious virus, with over 370 million clinically detected cases and over 5.6 million deaths (https://www.worldometers.info/coronavirus/; https://covid19.who.int/; accessed on 28 January 2022). The virus is still evolving and variants with new mutations are being discovered periodically [3].

### 1.2. Structure of SARS-CoV-2

The word corona is derived from the Ancient Greek (korōnè) and Latin (corona) meaning “garland” or “wreath”, or “crown”. In micrographs, the club-shaped glycoprotein spikes on the surface of coronaviruses give the appearance of a radiate crown, hence the name. SARS-CoV-2 belongs to the betacoronavirus genus of the order *Nidovirales*, family *Coronaviridae* and consists of positive-sense single-stranded RNA (+ssRNA) encapsulated with a membrane envelope. The four main structural proteins, namely spike (S) glycoprotein, envelope (E) glycoprotein, nucleocapsid (N) phosphoprotein, and membrane (M) proteins are encoded in the SARS-CoV-2 viral genome [7,8]. SARS-CoV-2 shares approximately 79.5% genomic homology with SARS-CoV [9]. A unique property of the SARS-CoV-2 versus other coronaviruses is the longer length of the spike protein, which has been suggested to contribute to its higher transmissibility [10]. 

### 1.3. Replication of SARS-CoV-2

SARS-CoV-2, as its predecessor, uses spike glycoproteins on its surface to bind to the angiotensin-converting enzyme 2 (ACE2) receptor on the plasma membrane of the mammalian host cells. It then uses serine protease transmembrane protease serine 2 (TMPRSS2) to prime the spike [11]. Receptor-mediated endocytosis guides the virus entry into the cell where it releases its +ssRNA, which multiplies using the cellular machinery, and, eventually, daughter nucleocapsids are released by exocytosis.

### 1.4. Organs Targeted by SARS-CoV-2

Along with pulmonary manifestations, SARS-CoV-2 virus also affects the heart, gastrointestinal system, liver, kidney, and the central nervous system (CNS), thus causing multiorgan failure. The most common symptoms of COVID-19 are fatigue, dry cough, and fever. Other comparatively less common symptoms include: nasal and chest congestion, conjunctivitis, headache, muscle or joint pain, skin rashes, nausea or vomiting, diarrhea, or chills. Neurological manifestations of COVID-19 (frequently referred to as neuro-COVID or nCoV [12,13]) are acute encephalitis, encephalopathy, ataxia, tremors, stroke (hemorrhagic or ischemic), hyposmia/anosmia, hypogeusia/dysgeusia/ageusia, Guillain-Barre-like syndrome, peripheral neuropathy and myopathy, delirium, nerve damage, irritability, or dizziness, anxiety, depression, fatigue and sleep disorders (https://www.who.int/COVID-19; https://www.ninds.nih.gov/Current-Research/Coronavirus-and-NINDS/nervous-system; accessed on 28 January 2022 [14,15,16,17]). Occurrence of these symptoms suggests an involvement of the nervous system [18,19,20,21,22]. 

There is a higher prevalence of the CNS complications in patients with severe disease. We discuss the progress made in investigating the repercussions of SARS-CoV-2 infection in the following text. Direct neuroinvasion of SARS-CoV-2 as well as CNS damage attributable to hyperinflammatory responses are discussed. Finally, we describe the potential of SARS-CoV-2 infection to incite or accelerate neurodegeneration in surviving patients and some of the promising therapeutic approaches.

**Table 1:** Therapeutics tested against COVID-19.

S. No.	Drugs/Therapies Tested	Mechanism/Site of Action	State of Success against COVID-19	References
Antivirals				
**1**	Remdesivir	A nucleotide analogue that inhibits the RNA-dependent RNA polymerase (RdRp) of coronaviruses including SARS-CoV-2	Shortened the time to recovery along with lower incidence of serious adverse events due to respiratory failure; improved survival but did not affect viral clearance	[23,24,25,26]
**2**	Lopinavir–ritonavir	The enzyme 3-chymotrypsin-like protease (3CLpro) plays a crucial role in processing the viral RNA. As a protease inhibitor lopinavir–ritonavir inhibits the action of 3CLpro, thereby disrupting the process of viral replication and release from host cells	No benefits observed	[27]
**3**	Favipiravir	An RdRp inhibitor, the active form of this prodrug acts as a substrate for the RdRp enzyme and gets incorporated in the viral RNA strand, preventing further extension	No benefit. Excessive ferritin forms a complex with favipiravir, thus reducing favipiravir levels in blood in moderate-to-severe disease. On the other hand, high levels of favipiravir and its inactive metabolite M1 inhibit the organic anion transporters in the kidneys resulting in enhanced reabsorption and reduced excretion of uric acid, thus increasing its concentration in blood	[28,29,30,31,32,33]
**4**	Favipiravir in combination with hydroxychloroquine	Inhibition of RdRp and viral binding to host membrane	One trial is underway, while another found efficacy in treatment	[34,35]
**5**	Chloroquine	An antimalarial, inhibits the action of heme polymerase in malarial trophozoites, preventing the conversion of heme to hemozoin. Interferes with virus binding to the host membrane by increasing pH and inhibiting ACE2 receptor	No beneficial effects	[36]
**6**	Hydroxychloroquine	An analogue of chloroquine, used to treat autoimmune diseases in addition to malaria. Mechanism of action similar to chloroquine	Did not affect viral clearance; no beneficial effects	[26,36]
**7**	Hydroxychloroquine in combination with azithromycin	Azithromycin is an antibiotic	Combination of hydroxychloroquine and azithromycin reduced viral load	[37,38,39,40]
**8**	Intravenous immunoglobulin (IVIg immunotherapy)	IVIg is a blood preparation isolated and concentrated from healthy donors mainly consisting of IgG. High-dose IVIg could modulate the activation of cytokine network, neutralize autoantibodies, and regulate proliferation of immune cells	In patients with severe disease, reduction in mortality was seen; in patients with non-severe COVID-19, no benefit was observed	[41,42,43,44]
**9**	Convalescent plasma (immunotherapy)	Passive immunization approach using antibodies from survivors	Effective supplementary treatment if applied early in the disease course	[45,46,47]
Steroids/anti-inflammatory compounds				
**10**	Dexamethasone (9α-fluoro-16α-methylprednisolone)	A glucocorticoid that increases the production of anti-inflammatory compounds	In hospitalized hypoxic COVID-19 patients, lower mortality was observed; another study is ongoing	[48,49]
**11**	Methylprednisolone	A synthetic glucocorticoid, with anti-inflammatory and immunosuppressive effects	Produced better results than dexamethasone; better clinical outcome, i.e., laboratory markers of severity (CRP, D-dimer and LDH), and shorter recovery time, was observed with methylprednisolone, which has been attributed to its higher lung penetration compared to dexamethasone; reduced mortality	[50,51,52,53]
**12**	Anakinra	A recombinant form of human interleukin-1 receptor antagonist (IL1R)	Safe and might be associated with reductions in both mortality and need for mechanical ventilation	[54]
**13**	Anakinra in combination with methylprednisolone	Anti-inflammatory	Risk of death was significantly lower for treated patients	[55,56,57]
Janus kinase inhibitors				
**14**	Ruxolitinib	Inhibitor of Janus kinases (JAK) 1 and 2, anti-inflammatory	Decreased the time on mechanical ventilation, hospitalization time, the need for vasopressor support, and decreased mortality and improved lung congestion. Phase III trial conducted by Novartis did not observe these beneficial effects	[58,59,60]
**15**	Baricitinib	Inhibitor of JAK, anti-inflammatory, and reduces receptor-mediated viral endocytosis	A phase I/II clinical trial is under way	[61]
**16**	Baricitinib (in combination with Tocilizumab and Corticosteroids)	JAK inhibitor	The addition of baricitinib did not substantially reduce mortality in hospitalized patients with COVID-19. Combination of baricitinib with corticosteroids was associated with greater improvement in pulmonary function	[62,63]
**17**	Baricitinib, ruxolitinib, tofacitinib	JAK/STAT inhibitor	Reduce excessive inflammation	[64,65]
Monoclonal antibodies against SARS-CoV-2				
**18**	Bamlanivimab	Monoclonal antibody treatment providing immediate, passive immunity	Accelerated the natural decline in viral load over time	[66]
**19**	Bamlanivimab in combination with etesevimab	These antibodies attach to the spike protein of SARS-CoV-2 at two different sites, preventing its entry into the cells	Statistically significant reduction in SARS-CoV-2 viral load	[67,68]
**20**	Casirivimab in combination with imdevimab	Bind to different sites on the receptor binding domain of the spike protein of SARS-CoV-2, blocking its attachment to the human ACE2 receptor	In high-risk patients, this treatment significantly reduced rate of hospitalization	[69,70,71]
Therapeutic antibodies targeting inflammatory cytokines				
**21**	Tocilizumab	Monoclonal antibody against interleukin-6 (IL-6) receptor	Reduction in mortality, intubation	[72,73]
**22**	Clazakizumab, olokizumab, siltuximab	Monoclonal antibody against IL-6, IL-8	Similar effects in diminishing leukocyte	[74,75,76,77]
**23**	Levilimab, sarilumab	Monoclonal antibody against IL-6R/gp130	Sustained clinical improvement	[78,79,80]
**24**	Canakinumab	Monoclonal antibody against IL-1β	Favorable prognosis compared to standard of care	[81,82]
**25**	Guselkumab, risankizumab, ustekinumab	Monoclonal antibody against IL-12/IL-23	Protects against COVID-19 in rheumatological patients	[83,84,85]
**26**	Ixekizumab, secukinumab	Monoclonal antibody against IL-17A	Beneficial effects of inhibiting IL-17	[86,87,88]
**27**	Emapalumab	Monoclonal antibody antagonist of interferon IFN-γ	Protects against cytokine storm resistant to anakinra, tocilizumab, and JAK inhibitors	[89]
**28**	Infliximab, adalimumab	Monoclonal antibody against tumor necrosis factor (TNF-α)	Facilitated clinical recovery in severe and critical cases	[90,91]
**29**	Gimsilumab, lenzilumab, otilimab, TJ003234	Granulocyte-macrophage colony-stimulating factor (GM-CSF) neutralization	Safe and associated with faster improvement in clinical outcomes	[92,93,94,95]
**30**	Namilumab	Monoclonal antibody against GM-CSF	Reduction in inflammation	[96]
**31**	Mavrilimumab	Monoclonal antibody against GM-CSF receptor	Improved clinical outcomes	[97,98]
Other compounds				
**32**	Dapansutrile	Selective and orally active NLRP3 inflammasome inhibitor	Clinical trials ongoing	[99]
**33**	Etanercept	Tumor necrosis factor receptor (TNFR) inhibitor	Protects against evolution to more severe disease	[100,101]
**34**	Melatonin	Blocks the activity of cluster differentiation 147 (CD147)	Has anti-inflammatory, anti-oxidant activities	[102,103,104]

## 2. SARS-CoV-2 and the Nervous System

SARS-CoV-2 is suspected to be a neurotropic virus, and there is evidence both in favor of and against this supposition. A broad organotropism of SARS-CoV-2 RNA was shown to occur in autopsied tissues including human brain [105,106]. SARS-CoV-2 was shown to infect and replicate in cells of neuronal origin [107]. Using induced pluripotent stem cells (iPSCs)-derived human neural progenitor cells (hNPCs), neurospheres, and brain organoids, Zhang et al. showed that these models expressed ACE2 and other key coronavirus entry-associated proteases, and were permissive to SARS-CoV-2 infection and supported productive virus replication [108,109]. High-titer anti-SARS-CoV-2 antibodies were found in the serum and cerebrospinal fluid (CSF) of patients with encephalopathy and in comatose patients [110]. SARS-CoV-2 was also shown to disrupt the blood–CSF barrier function [111,112,113]. In electron micrographs of autopsied frontal lobe tissue, SARS-CoV-2 viral-like particles were observed in neural and capillary endothelial cells [114]. SARS-CoV-2 has been detected in brain tissue, [106,115,116] including SARS-CoV-2 viral RNA transcripts [105,117,118], and viral proteins in the epithelial cells within the olfactory bulb [119]. However, on the other hand, studies showed that SARS-CoV-2 does not replicate in the CNS, and its CNS effects may be attributable to the immune responses that are triggered by the infection [120,121].

**Figure 1:**
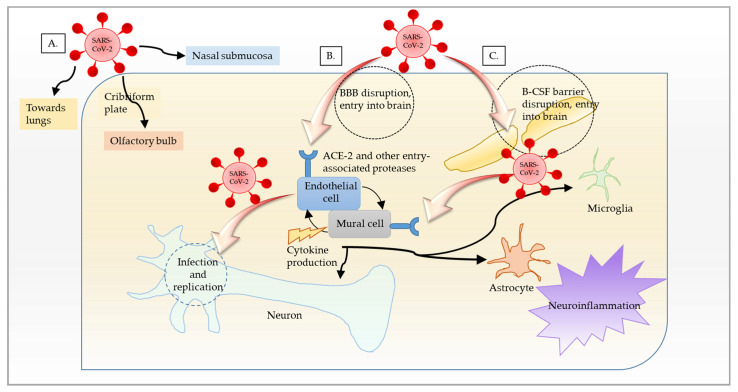
Schematic representation of SARS-CoV-2 entry routes. (**A**) SARS-CoV-2 enters the nasal cavity via droplets. It subsequently enters the blood through nasal submucosa. It may further obtain access to the olfactory nerves and, thus, the olfactory bulb by moving upstream. SARS-CoV-2 enters the lungs, crosses the thin alveolar membrane, and enters the blood to access all organs, including the brain. (**B**) SARS-CoV-2 binds to ACE2 receptor and gains entry into endothelial cells, infects, and replicates in cells of neuronal origin, leading to inflammation and opening of the BBB. Inflammation then spreads to vascular mural cells and other brain cells, such as microglia and astrocytes. The resulting alteration in neuronal function and inflammation results in encephalopathy in COVID-19. (**C**) Another possible way that SARS-CoV-2 could gain entry into the brain is through blood–CSF (B–CSF) barrier by binding to the ACE2 receptor in choroid plexus epithelium.

It may not proliferate there, but there is plenty of evidence that SARS-CoV-2 does reach and infect individual mature neurons as mentioned in the preceding paragraph and shown quite comprehensively by a couple of recent studies [118,122]. The key question is then, how does the virus reach the CNS? A few putative sites have been proposed for the entry of SARS-CoV-2 in the CNS: (1) through damage to the blood–brain barrier (BBB) epithelial cells and leukocyte migration across the BBB (hematogenous route), (2) neuronal retrograde pathways (active axonal transport), and (3) through the olfactory bulb (Figure 1) [123,124,125,126,127].

The S protein was observed in the cytoplasm of endothelial cells that had tested positive for SARS-CoV-2 RNA [118]. The RNA was also detected in the olfactory mucosa and in the neuroanatomical areas receiving olfactory tract projections, suggesting neuroinvasion of SARS-CoV-2 via axonal transport [118]. Direct evidence for the entry of the S1 subunit of SARS-CoV-2 spike protein was shown in mice that were injected intravenously with radioiodinated S1 (I-S1) [122]. In these mice, the I-S1 was shown to cross the BBB by adsorptive transcytosis and gain entry into various brain regions and parenchymal brain space. Moreover, intranasally-administered I-S1 was also found to reach all the brain regions examined [122]. These observations are important in view of the theory that S1 protein may shed from SARS-CoV-2 and enter the brain to elicit the same responses as the whole virus itself. Thus, it may alter the same factors and inflame the brain from inside out, in turn, making the brain more susceptible to further injury. Furthermore, the detection of the SARS-CoV-2 RNA in other brain regions, such as the cerebellum, that have no direct connection to the olfactory mucosa, suggests other parallel routes of infection, such as leukocytes carrying SARS-CoV-2 migrating across the BBB or viral entry via CNS endothelia. This assumption is supported by positive immunoreactivity to the SARS-CoV-2 S protein in cerebral and leptomeningeal endothelial cells [118]. The viral entry points in the CNS are further discussed below.

### 2.1. ACE2 Expression in Brain

The physiological function of the metalloproteinase ACE2 is to cleave Angiotensin-II into the vasodilator peptides Angiotensin-(1–7), and act as a central element in coordinating the effects of these peptides including neuroprotection [128]. In the context of COVID-19, as mentioned above, the ACE2 receptor aids SARS-CoV-2 entry into the cell. This fact has sparked renewed attention to the ACE2 expression in the nervous system. Indeed, several studies have shown that apart from the vascular and airway epithelia, lung parenchyma, kidney, and small intestine, ACE2 expression occurs in human and mouse brains, predominantly the cardiorespiratory neurons of the brainstem, as well as in non-cardiovascular areas such as the motor cortex and raphe [129,130,131,132,133,134,135]. ACE2 is ubiquitously present in brain vasculature, in astrocytes, in key components of BBB, and in discrete neuronal groups [136]. Low levels of ACE2 receptor expression were shown in neuronal and glial cells in the human CNS [137]. In brain cells expressing ACE2 receptors, neuroinvasion of SARS-CoV-2 is presumed to occur primarily via the olfactory bulb causing neuronal death in mice [138,139]. The dependency of SARS-CoV-2 infection on the ACE2-receptor was shown via lentiviral overexpression of ACE2 in endothelial cells, and via uptake of the radioactively-labeled S1 subunit of spike protein in the brain [122,140]. Previously, the brain was shown to be the major target organ for SARS-CoV infection in mice transgenic for ACE2 receptor, where the virus entered via the olfactory bulb and produced rapid, transneuronal spread to other regions of the brain. Neuronal demise, primarily of those located in the cardiorespiratory centers in the medulla, was proposed as the main cause of death of the animal, and only the absence of ACE2 receptors prevented the severe disease symptoms [123]. These observations, combined with the fact that both the ACE2 expression and SARS-CoV-2 infection are detected in the neurons of the primary respiratory and cardiovascular control center of the brainstem, indicate a CNS-mediated pathway in the respiratory or cardiac insufficiency observed in COVID-19 patients [118,138,141]. Another receptor that has been implicated in mediating SARS-CoV-2 entry through binding to the S protein, is a type II transmembrane glycoprotein cluster differentiation (CD)147. Significant expression levels of ACE2, CD147, and serine protease TMPRSS2 were detected in human and mouse brain cell lines, and in different brain regions of mice [142]. It is of interest to note here that several previously identified ACE2 inhibitors are currently being repurposed to treat COVID-19 and need further verification from clinical trials regarding their efficacy [143]. However, it is yet to be conclusively determined whether or not patients with hypertension and other cardiovascular comorbidities on long-term therapy with ACE inhibitors or angiotensin receptor blockers (ARBs) are at higher risk of poor outcomes from COVID-19 [144,145,146].

### 2.2. Cytokine Storm and Leaky BBB

Apart from the direct infection of CNS by SARS-CoV-2, systemic inflammation is also proposed to result in neurological manifestations seen in COVID-19 (neuro-COVID or nCoV) [147,148]. Cytokine storm is an acute systemic inflammatory syndrome that occurs when there is an overwhelming increase in proinflammatory circulatory cytokine levels, such as those observed in certain cases of COVID-19, (Interleukin (IL)-1β, -2, -4, -6, -7, -8, -9, -10, -18, granulocyte-macrophage colony-stimulating factor (GM-CSF), interferon γ-induced protein (IP)-10, monocyte chemoattractant protein-1 and -3, macrophage inflammatory protein 1α, cutaneous T cell-attracting chemokine, interferon-γ (IFN-γ), and tumor necrosis factor-α) [148,149,150,151,152]. This further leads to hypercoagulability. Considering this, several anti-inflammatory drugs have been repurposed to prevent cytokine storm in COVID-19 (Table 1). Inflammation and a hypercoagulable state are associated with acute cerebrovascular disease in COVID-19 [153,154]. The sequence of events ultimately results in a profound loss of T cells in COVID-19 patients. The systemic inflammation results in widespread organ damage followed by neurological symptoms. Studies have suggested that the endothelial activation and BBB disruption caused by peripheral inflammation results in astrocyte and microglia activation, which, in turn, produce oxidative stress and neuroinflammation [155]. Several cytokines are known to be transported across the BBB from blood to brain [156]. In addition, SARS-CoV-2 directly infects the immune cells and, at the same time, makes the BBB physiologically “leaky”, resulting in an exaggerated immune response and inflammation of the brain [124,138]. Using intranasal administration of the SARS-CoV-2 in animals, Zhang et al. showed that the virus damages the BBB integrity by damaging basement membrane, followed by increased viral loads in brain tissue and the activation of neuroinflammatory responses [157]. Thus, both the cytokine storm as well as a direct viral interaction seem to cause a leaky BBB, thrusting the peripheral immune cells inside the brain and aggravating the neurological damage. 

## 3. COVID-19 and Neurodegeneration

Neurodegeneration is characterized by selective and progressive loss of neurons in the CNS, peripheral nervous system, or both. Neuronal demise is a multifactorial phenomenon arising from a person’s genetic predispositions or metabolic/environmental risk factors. With changes in diet, life style, and environment, and with the increase in life expectancies, the epidemic of neurodegenerative diseases is on the rise. Advanced age is the major risk factor associated with some of these diseases including sporadic cases of Alzheimer’s disease (AD), Parkinson’s disease (PD), other dementias, various tauopathies, etc. All of these diseases are associated with increased neuroinflammation and oxidative stress, as well as mitochondrial dysfunction. Due to the overlapping CNS effects, it has been speculated that COVID-19 may initiate or worsen neurodegenerative conditions in surviving patients [158,159,160]. There is especially greater concern in patients that develop the so-called long COVID, with a broad range of symptoms that do not resolve over an extended period of time [21,161]. Chronic systemic inflammation may leave detrimental impacts on the brain, particularly resulting in sustained activation of microglia and astrocyte subtypes [162]. In severe cases of COVID-19, cytokine storm occurs and high levels of proinflammatory cytokines are known to cause cognitive decline including difficulties with concentration, memory, fatigue, and/or executive function [163,164,165]. Exaggerated systemic- and neuroinflammation may alter the course of or precipitate neuropathology in neurodegenerative diseases [166,167]. Based on their previous studies and others, Heneka et al. have proposed that SARS-CoV-2 infection results in NOD-, LRR-, and pyrin domain-containing protein 3 (NLRP3) inflammasome activation which, in turn, produces impaired clearance and pathological accumulation of neurodegeneration-associated peptides such as amyloid β (Aβ) and tau [163,168,169,170]. Using 3D human brain organoids, Ramani et al. showed that SARS-CoV-2 preferably targets neurons, causing mislocalization of tau from axons to soma, tau hyperphosphorylation, and, ultimately, neuronal death [171]. An analysis of single-nucleus transcriptomic profiles revealed significant overlaps between COVID-19, and neuroinflammation and brain microvascular injury pathways that are implicated in AD [172]. This study further unveiled alterations of multiple common factors involved in cognitive impairment observed in COVID-19 and AD. There is evidence that SARS-CoV-2 can aggravate the clinical presentations of PD [173,174,175,176] and cause de novo problems with gait, movement, coordination, and balance [177,178,179,180]. The lasting impacts of SARS-CoV-2 infection on microglial plasticity and its contribution to the pathophysiology of post-COVID-19 neurological sequelae and disorders, including PD, were comprehensively discussed in a recent review [181]. In line with the above mentioned findings, cognitive impairment and dysexecutive syndrome consisting of inattention, disorientation, or poorly organized movements in response to command were observed to be persistent several months after the infection in COVID-19 survivors [182,183].

In addition to the direct adverse effects of SARS-CoV-2, the restrictive measures enforced due to COVID-19 such as home confinement and social distancing negatively impacted the general health and well-being of all individuals causing depression, anxiety and stress, and worsened the neuropsychiatric outcomes for AD patients [184,185,186,187,188]. Studies have shown that isolation and reduction in mobility increased psychological stress and produced an impairment of motor and nonmotor symptoms in PD patients [189,190,191,192]. There is also a prevalence of post-traumatic stress disorder (PTSD) among COVID-19 survivors and care givers [165,193,194,195,196].

It is clear from the preceding discussion that there is a risk for development/worsening of neurodegeneration among COVID-19 survivors. Neurons have a high metabolic demand and limited regenerative capacity, and they depend heavily on energy produced in mitochondria for their adequate functioning and survival. Therefore, we next focus on the mechanistic aspects of mitochondrial alterations and mitochondria-targeted therapeutic opportunities in COVID-19.

### 3.1. Oxidative Stress, Dysregulation of Iron Homeostasis, and Mitochondrial Dysfunction

Hyperferritinemia, also known as iron dysregulation, occurs in exaggerated inflammatory states and was shown to be associated with increased mortality in COVID-19 [149]. Iron dysregulation produces reactive oxygen species (ROS), enhances oxidative stress and results in iron-dependent cell death, termed ferroptosis [197,198]. The mitochondria are the focal point of cellular energy metabolism as well as oxidative homeostasis. Iron overload leads to abnormal mitochondrial morphology, resulting in a fragmented architecture, overproduction of mitochondrial ROS, alteration of mitochondrial membrane potential, elevated mitochondrial lipid peroxidation, and further accumulation of mitochondrial iron. Ferroptosis works by compromising mitochondrial functionalities by restricting mitochondrial oxidative phosphorylation and anti-oxidant response, and the mitochondrial DNA (mtDNA) is vulnerable to iron deposition [199,200,201,202]. An involvement of other organelles, including the endoplasmic reticulum, Golgi apparatus, and lysosomes, has been suggested in ferroptosis, although most studies emphasize a multifaceted regulation of ferroptosis by mitochondria [203]. Using computational modeling, SARS-CoV-2 genomic and subgenomic RNA was shown to localize in the host mitochondrial matrix and nucleolus [204]. Wu et al. [204] further showed that the 5′- and 3′-untranslated regions of SARS-CoV-2 contained the mitochondrial localization signals. SARS-CoV-2 disrupts the host ubiquitin system to inhibit the functions of IFN-I, which serves as the first response that is triggered upon encounter with the virus to stage the uprising of immune cells to the so-called antiviral state [205]. SARS-CoV-2 also targets a mitochondrial deubiquitinase involved in mitochondrial homeostasis and mitophagy, thus relaying the possibility that SARS-CoV-2 might affect mitochondrial function by altering ubiquitination [206]. An alternative open reading frame (Orf9b) within the nucleocapsid gene of SARS-CoV-2 was shown to interact with the outer mitochondrial membrane and inhibit IFN-I production [207,208]. Orf9b also directly interacts with a component of the translocase of the outer mitochondrial membrane (TOM70) and thus interferes with the import of the mtDNA transcription factor A (Tfam) [207,208,209]. SARS-CoV-2 suppresses mitochondrial respiratory chain complex I expression and impairs mitochondrial energy production [209]. Altered bioenergetics and mitochondrial dysfunction was observed in peripheral blood monocytes in patients with COVID-19 pneumonia [210]. A link between SARS-CoV-2 infection, mitochondrial dysfunction, and inflammation, leading to persistent brain-fog, has been suggested [211,212]. The activation of the oxidative stress pathways coupled with mitochondrial dysfunction and innate immunity further exacerbate proinflammatory responses and thus play a major role in COVID-19 pathogenesis and severity [213,214,215]. Mitochondrial dysfunction and hallmark features of ferroptosis (including iron deposition, GSH depletion, and elevated lipid peroxidation, i.e., oxidative stress) are consistently observed in neurodegenerative diseases, including AD and PD [216,217,218].

### 3.2. Therapeutic Prospects

The transcription factor nuclear factor erythroid 2-related factor 2 (Nrf2) plays pivotal roles in oxidative defense and iron metabolism [219]. Nrf2 pathway is suppressed in biopsies obtained from COVID-19 patients [220]. Activators of Nrf2 help prevent ferroptotic death [221] and have been explored as potential therapeutic agents against COVID-19 [220,222].

Another pathway of interest from a therapeutic perspective and one that works upstream of Nrf2 is the peroxisome proliferator-activated receptor-γ (PPAR-γ) coactivator-1α (PGC-1α) pathway. PGC-1α is a widely studied master regulator of mitochondrial bioenergetics and anti-oxidant defense, and its impaired expression and/or function has been associated with various neurodegenerative disorders as well as metabolic diseases [223,224]. PGC-1α orchestrates the myriad of biological pathways via its interaction with and regulation of the expression of a variety of transcription factors including nuclear respiratory factor-1 (NRF-1), PPARs, 5′-adenosine monophosphate (AMP)-activated protein kinase (AMPK), Sirtuins, and Nrf2 [225,226,227]. AMPK is a key energy sensing molecule that modulates cell growth and proliferation, and is involved in several other important functions of the cell such as, autophagy, stress responses, mitochondrial homeostasis, and host immune function. AMPK phosphorylates and enhances the expression of ACE2. SARS-CoV-2 has been shown to interfere with AMPK functions [228]. Sirtuin 1 (SIRT1) activates PGC-1α through its deacetylation, and expression of SIRT1 was significantly decreased along with increased concentrations of plasma proinflammatory cytokines in peripheral blood mononuclear cells from COVID-19 patients [229]. Studies have pointed towards prominent roles of PPAR-α and PPAR-γ in inflammation and metabolic regulation [230]. PPARs have an established immune-modulatory role, and the use of PPAR as adjuvants to vaccines for COVID-19 has been prescribed due to their role in enhancing immunologic memory [231].

Therapeutic targeting of PGC-1α through small molecules or physical exercise is of interest because of three main reasons: firstly, it integrates all the above mentioned factors that are affected in COVID-19; secondly, due to its multifaceted roles in anti-inflammatory and anti-oxidant pathways as well as in improving mitochondrial function, all of which are disrupted in COVID-19; thirdly, it shows promise in neuroprotection [232,233,234,235,236,237,238]. Increased PGC-1α expression was shown to reduce NLRP3 inflammasome activation, proinflammatory cytokine production, and decrease neuroinflammation and depression-like behavior [239,240]. Several compounds have been identified in other models of neurological damage that reduce neuroinflammation and oxidative damage through AMPK/PGC-1α– or SIRT1/PGC-1α–mediated pathways and improve cognitive outcomes [241,242,243,244]. Melatonin is a hormone involved in control of the sleep–wake cycle and has anti-inflammatory, anti-oxidative, and mitochondrial protective properties. It increased PGC-1α, Nrf2, and other mitochondrial genes, and restored mitochondrial structure and function in in vitro models of AD [245]. The use of melatonin has been suggested as a potential adjuvant for COVID-19 [246,247,248]. A recent study showed that the combination of oral melatonin tablets with standard care substantially improves sleep quality and blood oxygen saturation in hospitalized COVID-19 patients [249].

In recent years, there has been increasing data for the effectiveness of natural extracts such as polyphenols, flavonoids, and saponins as potent anti-oxidant and anti-inflammatory agents (reviewed in: [250]). Phytochemicals such as resveratrol, curcumin, mulberry extract, green tea polyphenols, etc., have been shown to energize mitochondria through the SIRT1/PGC-1α, AMPK/PGC-1α, or Nrf2/PGC-1α axis, and exert neuroprotective effects and improve overall health [251,252,253,254,255]. The therapeutic potential of thymoquinone, a component of *Nigella sativa*, against COVID-19 is worthy of further investigation due to multiple evidence of this compound’s efficacy in targeting the AMPK/PPARγ/PGC-1α and Nrf2/heme oxygenase (HO-1) pathway and in preventing SARS-CoV-2 entry into the cells [256,257,258]. Moreover, several plant-based compounds have antimicrobial activities, and some of the polyphenolic compounds, including flavonoids, terpenoids, hydrolysable tannins, etc., have potential inhibitory properties against ACE2 receptors [143,259]. Using molecular docking analysis, bioactive ligands were identified from medicinal plants that inhibited SARS-CoV-2 viral replication and transcription [260,261]. Such phytochemicals interrupt the viral entry into the host cell by interaction with ACE2, TMPRSS2, and spike glycoprotein and, consequently, halt viral replication, pathogenicity, and transmissibility. Many of the aforementioned compounds are already approved for use in other diseases. Suitable clinical trials ought to be conducted to investigate the neuroprotective effects of potential therapies, including drugs and physical exercise in patients with confirmed disease progression in COVID-19. The primary outcome measure would be neurodegeneration, which could be measured as whole brain atrophy on magnetic resonance imaging (MRI). Secondary outcome parameters may include other biomarkers associated with neurodegeneration, for example, regional brain atrophy, lesion load, white matter integrity, resting state functional connectivity, blood biomarkers, and physical, neuropsychiatric, and cognitive measurements. We briefly discuss some of the biomarkers currently being used and newer biomarkers that are being discovered and developed in COVID-19 and neuro-COVID.

### 3.3. Biomarker Identification and Development

Because of the novel and abrupt nature of the disease, several radiographic and laboratory parameters have been tried and tested to predict the progression of COVID-19. These biomarkers help clinicians in providing better prognostics and management of patients with the disease. One of the widely used biomarkers highly associated with disease progression in COVID-19 in both acute and mild cases is increased cytokine levels in blood [262]. Both CT scan scores and the elevated serum levels of inflammation and tissue damage markers, such as IL-6, IL-8, interferon-γ (IFN-γ), C-reactive protein (CRP), and lactate dehydrogenase (LDH), are used to monitor the condition of patients [263]. IL-6 was shown to be one of the more robust prognostic markers of survival, even more so than CRP, D-dimer, procalcitonin (PCT), lymphocyte, and ferritin [264,265]. TNF-α, known to contribute to organ damage, is a strong predictor of a poor outcome. At the same time, a combination of hepatocyte growth factor (HGF), a pleiotropic cytokine, and C-X-C motif chemokine ligand 13 (CXCL13), a proinflammatory chemokine, was identified as an important predictor of severity of disease and death as an outcome [266]. Another study reported increased levels of IL-4 in the plasma of COVID-19 patients, which may indicate an adaptive response to neuroinflammation, and together with IL-6 levels may be used to predict outcome in neuro-COVID [13]. This study also analyzed the cargo of the neuronal-enriched extracellular vesicles (nEVs, also known as neuron-derived exosomes (NDEs)), which have been previously described as biomarkers in human immunodeficiency virus (HIV)-related cognitive impairment, AD, and traumatic brain injury (TBI) [267,268]. Interestingly, they found increased levels of inflammatory and neurodegenerative proteins in the cargo of these nEVs, which, if persistent, may indicate synaptic disruption and neuronal damage reminiscent of AD, PD, and TBI [13]. Results from a very recent study seem to be in agreement with these findings as blood biomarkers of neuronal and glial degeneration were found to be elevated in hospitalized COVID-19 patients with new onset cognitive dysfunction (specifically, toxic-metabolic encephalopathy, TME) [269]. Specifically, they showed increased levels of total tau, neurofilament light chain (NfL), glial fibrillary acidic protein (GFAP), and ubiquitin carboxy-terminal hydrolase L1 (UCHL1) in the blood of neuro-COVID patients, which together indicated a profound neurological insult comparable to non-COVID patients with AD [269,270]. It is of great significance to note that these neurodegenerative biomarkers are also elevated after BBB disruption and were found to be associated with a higher risk of in-hospital death and reduced rates of discharge from hospital [269]. The discovery of such comparatively fewer invasive blood biomarkers for cognitive impairment could help in monitoring neuronal and other brain cell health in real time, and to determine treatment responses in neuro-COVID.

## 4. Conclusions

COVID-19 is fast turning into an endemic that the world population will have to live with over the next coming years. Currently, vaccination is our primary hope to protect us from the more severe consequences of this deadly disease, although some of the therapeutics also imparted improved clinical outcomes, as outlined in Table 1. SARS-CoV-2 has already taken a huge toll in terms of millions of deaths all over the world and long-lasting sequelae in COVID-19 survivors. Neurological consequences are being widely reported and mitochondrial involvement seems to be a crucial element in it. Targeting PGC-1α mediated pathways seems to be of benefit for the mitochondrial and neuronal health. We have discussed a few of the approaches above and many more are in development [271]. Direct mitochondrial transplantation from healthy cells was recently suggested as an innovative therapy in COVID-19 [272,273]. Although full of potential, further research in this approach is needed [274,275,276]. Recently, the U.S. Food and Drug Administration issued an emergency use authorization for Merck’s molnupiravir developed with Ridgeback Biotherapeutics, and also approved Pfizer Inc’s Paxlovid for the treatment of COVID-19. A number of other therapies are being tested at lightning speed, giving hope for a potential cure [277,278,279,280,281,282,283,284,285,286,287]. However, in the current environment, the potential of holistic approaches including diet, exercise, and a healthy lifestyle cannot be underestimated in keeping the brain and mind healthy, as well as to boost natural immunity, as this virus does not seem to be letting up in the near future.

## Data Availability

Not applicable.
